# Parent-Child Sex Communication Prompts, Approaches, Reactions, and Functions According to Gay, Bisexual, and Queer Sons

**DOI:** 10.3390/ijerph19010074

**Published:** 2021-12-22

**Authors:** Dalmacio D. Flores, Madelyne Z. Greene, Tamara Taggart

**Affiliations:** 1 Department of Family and Community Health, University of Pennsylvania, Philadelphia, PA 19104, USA; 2 School of Nursing, University of Wisconsin-Madison, Madison, WI 53705, USA; mgreene8@wisc.edu; 3 Department of Prevention and Community Health, George Washington University, Washington, DC 20052, USA; ttaggart@gwu.edu; 4 Department of Social and Behavioral Sciences, Yale School of Public Health, New Haven, CT 208034, USA

**Keywords:** HIV, STI, YMSM, sexual health, prevention

## Abstract

Minimal research on parent-child sex communication between parents and gay, bisexual, and queer (GBQ) adolescent sons prevents the formulation of interventions that would buffer or brake this youth population’s risks for HIV/STI. We sought to describe the perspectives of GBQ adolescent males on this process and the potential ways they think parents can address their sons’ informational needs, including countering youth access of sexually explicit media. We conducted 30 semi-structured interviews with GBQ male youth aged 15–20 years. Thematic and content analysis revealed four central themes: prompts and triggers, parents’ approaches, sons’ reactions, and the functions assigned to sex communication. Parents can be sources of reliable sexual health information and may be leveraged for future HIV/STI risk reduction work.

## 1. Introduction

Sexual minority adolescents are at elevated risk for negative health outcomes compared to their heterosexual counterparts. Of the estimated 46,000 cases of HIV diagnosed in 2017 in the U.S., the primary transmission mode (70% of cases) was male-to-male sex. Of these, 7700 infections (16.7%) were among adolescent gay and bisexual males between 13 and 19 years of age [1]. The sexual initiation patterns of young men who have sex with men indicate that many have early sexual debut including anal sex experiences [2]. Furthermore, young men who have sex with men are less likely to report receiving HIV education in public school settings, but more likely to report sexual risk behaviors than young heterosexual males [3]. To counteract the lack of sexuality-congruent resources, this population uses the Internet to accesses sexually explicit media (SEM) for information about sex [4,5]. 

Sexual minority adolescents and young men in the U.S. often turn to SEM online to anonymously explore their emergent attractions, behaviors, and identities [5]. Gay and bisexual men view SEM online at higher rates than heterosexual men [4]. Although SEM can provide relevant information about the mechanics of same-gender sexual intercourse [6], inaccurate portrayals and normalization of risky sexual behavior may also place this youth population at risk for HIV/STI infections [7]. For example, SEM consumption has been associated with engaging in sexual risk behaviors including unprotected anal intercourse [8]. Indeed, for sexual minority adolescents, the lack of accurate and accessible HIV and sexuality information likely contributes to high HIV risk behaviors during sexual initiation [9], particularly in the Deep South and among gay youth and men of color [10,11]. However, when sexual minority adolescent males still reside at home, parents may be a proximal resource for HIV/STI prevention and other sex-related information [12].

### Parent-Child Sex Communication

Parents play a critical role in providing accurate HIV prevention and sexuality information to their child and have the potential to counter the negative effects of SEM. Sex communication among presumably heterosexual samples is associated with HIV-protective behaviors such as using condoms, resisting pressure to have sex, and accessing sexual health services [13,14]. However, American parents tend to think of their children as too young, regardless of age, to learn about sex and are reluctant to talk to their children about sex and safer sex behaviors [15]. When discussions about safer sex behavior occur, they have larger effects on girls than boys and with youth who have these discussions with mothers instead of fathers [16,17]. 

While much is known about the sex communication processes between parents and presumably heterosexual children, little is known about how these processes occur for families with gay, bisexual, or queer (GBQ) sons [18]. Recent positive shifts in social acceptance of lesbian, gay, bisexual, transgender, and queer (LGBTQ) individuals in the U.S. [19] have resulted in children coming out as LGBTQ at younger ages [20,21]. In these cases, parents have more opportunities to positively impact their child’s development through parent-child sex communication. For example, sexual minority males who have had more frequent talks about HIV with their parents also reported higher awareness of pre-exposure prophylaxis [22], the daily oral tablet for HIV prevention. However, lack of information on the nuances of parent-child sexual communication processes for GBQ youth has prevented the development of tailored, parent-driven HIV prevention interventions which might decrease GBQ youth’s engagement in HIV risk behaviors [23,24].

Research on sex communication between parents and GBQ adolescents and young men depicts a process fraught with awkwardness and fear. Parents of sexual minority youth have a minimal understanding of LGBTQ-specific issues despite concerns about their GBQ sons’ long-term sexual health [25]. Parents and gay sons mutually view these sex conversations as awkward [26] and parents who look for information that would be concordant with their GBQ sons’ questions and interests cannot find these resources online [27]. Despite these barriers, GBQ sons report a sense of obligation to their parents to stay healthy [28] and parents want their sons to grow up as healthy adults [29].

In the current study, we call on the “brake and buffer” hypotheses of parental sex communication, forwarded by Overbeek, van de Bongardt, and Baams [30]. The “buffer hypothesis” suggests that parents may buffer or mediate the *effects* of SEM consumption by “explaining and critically reflecting on” the content of the SEM that their sons consume. Alternatively, the “brake hypothesis” suggests that specifically tailored parent-child sex communication may *slow down* adolescents’ development toward increasing sexual behavior and SEM consumption. Affecting the use and effects of SEM consumption may be an especially important function of parent-child sex communication for young GBQ adolescent sons, given their early exposure to SEM and low access to affirming and relevant information about healthy sexuality in offline settings such as family, peers, or schools. The current study (1) examines the perspectives of GBQ sons on the initiation of—and their responses to—sex communication with parents, and (2) situates the processes and functions of this communication within the buffer or break discussion relative to SEM consumption. The examination of parent-child sex communication experiences from the perspective of these youth can assist in the development of parent-centered HIV prevention interventions that promote healthy sexual behavior among GBQ adolescents before lifelong behaviors begin to form.

## 2. Methods

Using a descriptive qualitative design, we recruited English-speaking, self-identifying gay, bisexual, and queer adolescent males aged 15 to 20 residing in North Carolina, who could recall at least one episode of parent-child sex communication. Flyers were distributed at gay-straight alliances in area high schools, LGBT student centers at universities, and non-profit organizations serving LGBT individuals. The primary recruitment venues were chosen as sexual minority youth, regardless of disclosure status about their sexual orientation to parents, frequent these spaces for social support. With many GBQ youth often coming out to friends first prior to sharing their sexual orientation with family, recruiting from these spaces allowed us access to a rich participant pool who could best describe how PCSC unfolds in their homes.

### 2.1. Data Collection

Participants completed semi-structured interviews in English approximately 60 to 90 min in length. Participants were asked to recall times that parents addressed sex with them. Probes were used to elicit further details about their reactions and thoughts on the sex communication process. Participants between 18 to 20 years old completed informed consent and those 15 to 17 years old signed assent forms. Because we aimed to include the experiences of GBQ males who had not disclosed their sexual orientation with their parents and potentially experiencing PCSC in real time, a waiver of parental consent was obtained from the Duke University IRB. All semi-structured interviews were conducted by the first author, a young gay-identifying male who received graduate-level training in qualitative data collection methods. Prior to the semi-structured interviews, participants completed a one-page demographic questionnaire. Data from the entire sample was collected in 2016. Further, to ensure that participant safety and mental health were prioritized during the interviews, we devised a rigorous protocol in the event of retriggering trauma or emotional distress [31].

### 2.2. Data Preparation and Analysis

Descriptive statistics were used to summarize sample characteristics. After each interview was conducted, the audio recordings were transcribed and verified for accuracy. Once initial participants were interviewed, content analysis was used with the assistance of NVivo 11 software (QSR International, Burlington, MA, USA)). Two members of the research team reviewed and coded the first 10 transcripts and compared notes about preliminary response patterns. This initial set of transcripts was coded both deductively and inductively based on sex communication literature and the emergent data. The lead author coded the transcripts in their entirety and the senior study member supervised and conducted spot checks to ensure quality and thoroughness. Initial response patterns guided the restructuring of the interview guide to more fully explore developing categories such as the nature of first exposure to SEM and the function of sex communication for sons. Twenty subsequent interviews were conducted until the study team agreed that saturation – the point when no new codes were being brought up in the interviews – was achieved. We identified four central themes on sex talk with parents from the perspective of gay, bisexual, and queer males: (1) prompts and triggers of sex communication, (2) parental approaches to communication, (3) sons’ reactions, and (4) the functions assigned by participants to the sex communication process. We relate our sub-themes to the buffer and brake hypotheses in light of participants’ reports of ubiquitous SEM consumption. Other dimensions of parent-child sex communication, such as the frequency of sex talks, parents’ knowledge of LGBT issues, and parental ratings as sex educators have been reported elsewhere [32,33].

## 3. Findings

### 3.1. Demographic Summary

Our sample included 30 GBQ males, with 23 identifying as gay, five as bisexual, and two as queer. Eleven (36.7%) were white, 10 (33.3%) were Latinx, four (13.3) were African American, four (13.3) were Asian American and one (3.3) identified as multiracial. The majority of the sample (*n* = 25; 83%) were 18 years or older, 26 (87%) of the participants had disclosed their sexual orientation to parents, and 19 (63%) were college students. The mean age of first same-sex attraction was 10.5 years (SD = 3.5 years), first exposure to SEM was 10.9 years (SD = 2.2 years), first time searching for GBQ-specific SEM was 13.5 years (SD = 2.0 years), first self-identification as GBQ was 14.7 years (SD = 2.2 years), and first disclosure as GBQ to another person was 15.4 years (SD = 2.6 years).

### 3.2. Conversation Prompts

There were several prompts or triggers that initiated health and sexuality discussions at home: when sons initiated the conversations by asking questions, when parents observed signs of sons’ physical maturation or social milestones, when family stories were being shared, and when parents capitalized on teachable moments to initiate talks about health and sexuality.

Son-initiated conversations. Prior to self-identification as GBQ, sex communication between parents and sons was mostly initiated by sons asking parents about sex-related topics. At very early ages, curiosity about human reproduction or “where babies come from” was the most cited question participants asked their parents. Participants also recalled seeking answers from parents about sex-related topics they overheard at school or with their peers.

“I overheard my sister and her friends so I asked my Mom, ‘What does third base mean?’ She was like, ‘Oh well, when you love someone and you’re doing stuff with them there’s different levels of what you do. The first one’s kissing and the second is touching and the third one is using your mouth…’”

(Amber, 15 years old, queer, White)

During high school and college (often after coming out), GBQ sons asked for parents’ opinions about relationship-related issues, including their views on same-sex marriages.

“I was reading young adult fiction in 6th grade and I remember a very oblique reference where the girl in the story yells at one of the characters for ‘going too fast.’ And I was like, ‘What does that mean? Is he kissing her too fast? What is that?’ So I asked [my parents] and my mom said something to the effect of, ‘It’s about sex.’”

(John, 18 years old, bisexual, Latino)

Physical maturation and social milestones. Parents’ perceptions that their child was approaching or had reached a maturational milestone (e.g., puberty) triggered parents to teach their sons about sex-related topics and human development.

“I was probably in sixth grade, and Dad came in my room and apparently thought I was ‘exploring.’ So later that night he came in and he basically explained to me what masturbation was since he thought I was old enough.”

(George, 19 years old, gay, White)

In some cases, parents provided books on what to expect during adolescence and followed up to see if sons had any questions. Others provided condoms and instructions on their use during penile-vaginal intercourse. Social milestones, such as sons transitioning to middle school or high school, preparations for the prom, or leaving home for college also prompted parents to initiate discussions on sexual behavior, though parents almost exclusively discussed sex between males and females.

“Two or three days before I left for college I could see my mom tensing up. She didn’t use the word condom or HIV or gay or anal or anything like that, but she said, ‘You know you have to be careful.’”

(Marcus, 18 years old, gay, White)

Sharing family stories. Some parents used family stories to prompt sex communication with sons and contextualize health-related lessons. These stories often highlighted the risks with engaging in sex such as a father’s story about getting an STI in college or an older sister’s pregnancy scare.

“The majority of sex information I got from my dad and his personal stories. He literally tells us everything that he’s done with girls, and all his college life, and all that. He’s very open with us now that we’re older. I remember the story of when he got the crabs from his roommate having sex on his bed…and how he got checked for STDs. He has a filter. He knows when to turn it on. He turns it off around his kids because he wants to share as much knowledge with us as he can.”

(Gauis, 18 years old, bisexual, Black)

After coming out, gay-specific stories involving extended family members and friends were also shared by parents, mainly to discuss concerns about acquiring HIV.

“One of my cousins has HIV. As a family we avoid talking about it—not because we’re ashamed but because it’s a sad talk to have. But sometimes you have to have that talk and my mom would be like, ‘Remember your cousin. Make sure you’re protected. Make sure you know that there are STDs out there. Make sure to know what the warning signs are of somebody who has STDs.’”

(Gauis, 18 years old, bisexual, Black)

Other teachable moments. For many of the participants, parents had initiated discussions by capitalizing on what they perceived as teachable moments. For example, viewing television content of a sexual nature was often followed by discussions about sex.

“We were watching something in the news, and we see a politician with a sex scandal. [My parents] gave their little spiel saying, ‘Hey, this is why you should save sex for marriage. Look at what happens when you don’t…’ They presented the fact that I shouldn’t have sex, and that was the conversation.”

(George, 19 years old, gay, White)

School-based sex education also triggered parental sex communication as most parents had to give consent for sons’ participation in these classes. Also, the discovery of sons’ online viewing of SEM also triggered discussions between parents and sons.

“She said she didn’t know why I was doing that [looking online for gay-themed SEM]. She didn’t understand why I had pictures of naked guys. I just told her I didn’t know, I just really didn’t know. And then she talked about when she was a little girl, she didn’t do that. And that was pretty much it. We didn’t really talk about it after that.”

(Tilapia, 19 years old, gay, Latino)

### 3.3. Parental Approaches

The participants described four distinct approaches or strategies used by parents to have conversations or respond to sons’ inquiries. Parents lectured, informed, bargained, or joked with their sons during sex talks.

Lecturing. Lecturing was the most commonly recalled parental approach and was marked by the unidirectional provision of information from parent to child. Topics of lecturing-style conversations included promoting abstinence, the implications of unplanned pregnancies or sexually transmitted infections, and the perils of unsupervised online engagement. These conversations underscored the consequences of sex. When parents used the lecturing approach, they were prescriptive in tone and sons felt they were expected to not question parental authority.

“I think he [my dad] asked me, ‘Do you know what gonorrhea is? Do you know what syphilis is?’ He said, ‘Your peepee is gonna have an infection, like a bacteria or fungus will grow’—very scary visuals, not very accurate, too. AIDS he didn’t explain in great detail but it was just, ‘Do you know what this is? It’s a disease that makes your peepee fall off.’”

(Ian, 20 years old, gay, Asian)

“When I had my first sexual experience at a summer camp I was afraid to talk to [my dad] about it. When he did hear about it, he said that it was completely inappropriate and that I need to promise him that nothing like that would ever happen again until I was 18 and out of the house.”

(Amareccio, 15 years old, bisexual, White)

Informing. Participants recalled parental approaches as sometimes being informative about sex-related issues they deemed essential for sons to know, which were mostly heteronormative in content. Parents easily delivered information about biological and developmental processes that were devoid of the personal or social implications of sex. When sons wanted to verify sex-related ideas they overheard outside the home, they found their mothers to be mostly open and conversational.

“She [Mom] covered almost the same things that my dad did, and then she went into detail about the female anatomy which was very disturbing for me at the time. She was talking about popping cherries and all that with virginity, and I was like, ‘Oh, okay.’ I did not know that happened. I didn’t want to know that happened…She was basically, ‘Don’t be scared. This happens because this is normal.’”

(Gauis, 18 years old, bisexual, Black)

In comparison, some fathers were business-like and used images and diagrams to explain concepts such as human reproduction.

“I asked my dad how babies were made and he just decided that he would tell me. My dad explained in a very scientific matter about what a penis and a vagina does and sperm and all this other stuff. He drew a diagram and whatever. I think I was around 7, and my stepmother strongly objected to him telling me about it, but he did it anyway. He thought it was fine to do so.”

(James, 19 years old, gay, Black)

Bargaining. Parents’ appraisal of sons’ current or imminent sexual behaviors sometimes resulted in a more conciliatory approach to the sex talk. Parents bargained with their sons after acknowledging the inevitability of engaging in sex.“ Once she [my mom] caught me masturbating. She was like, ‘You should try not to do it because it’s a sin. But if the alternative is you having sex with a girl then go ahead and do it.’” (James, 20 years old, gay, White)

Condom use was the most commonly cited topic that typified these pragmatic discussions. Whereas lecturing and informing approaches left little to no room for negotiation, a bargaining approach included parental openness to the possibility of sons having sex and therefore reducing their risks by discussing “safe sex.”

“They said, ‘Okay. Try not to have sex before marriage. But if you do, be safe about it.’”

(Bilbo, 18 years old, bisexual, White)

Joking. Multiple participants noted how approaching the sex talk with humor and jokes set a relaxed tone for sex communication. For example, when the participants were younger, they were teased by parents about which female playmate was their girlfriend or who of the opposite sex they would like to marry someday. Sons recalled parents employing jokes to minimize the chances that sons would become offended or find the conversation an intrusion of their privacy.

“When I was in fifth or sixth grade, my sister and her friend were joking about blowjobs, and I asked my mom what a blowjob was, and she told me. I said, ‘Mom, what’s a blowjob?’ and she laughed and went ‘Really? You don’t know?’ I think she said, ‘It’s when a girl sucks on a guy’s penis.’”

(David, 20 years old, gay, White)

Jovial sarcasm or teasing seemed to keep parents from overreacting to sons’ innocent queries, which in turn encouraged future conversations. Even when parents were pushing for details about their sons’ sexual lives, the deft use of humor dispelled tension and resulted in sons sharing more than they normally would. Off-color jokes made by parents were recalled after sons’ disclosure of their sexuality to acknowledge familial comfort about sons’ sexual orientation. According to sons, these good-natured jokes normalized their identities by showing that parents treated them like other children in the family through an equal chance at being affectionately teased.

Charles: Now [that we’re older], sex is such a casual topic in my family, it’s not censored anymore.

Interviewer: Really? Like what kind of conversations about it?

Charles: Ha, just like dirty jokes. That’s perfectly fine. My mom would get super upset, “You guys should not talk about that!” But my dad makes jokes and stuff like that. Now, sex is isn’t something that we should be ashamed of. It’s just something we laugh about… well actually the first thing my sister asked [after I came out] was “can I still make gay jokes?” and I’m like “Yes you can still make gay jokes!”…they love gay jokes (laughing). (Charles, 19 years old, gay, Latino)

### 3.4. Sons’ Reactions

Participants recalled sex communication with parents as generally awkward. Although they tended to be *compliant* with these talks, most participants’ initial reactions were negative, including feeling *mortified*, *dismissive*, *isolated*, and *offended*.

Compliant. A few participants saw the sex talk as a rite of passage that was important for parents to provide. Some sons acknowledged the inevitability of sex communication and were dutiful by playing along during these conversations.

“In the beginning of the conversations I pretended like I didn’t know anything because I wanted to be that good kid. ‘Cause I’m generally the good kid my parents think I am. So I said, ‘Oh, what is that?’”

(Ian, 20 years old, gay, Asian)

Participants recalled how they sat through admonitions to use protection because listening to parents was expected of them. Participants most often had this type of reaction when parents provided heteronormative information and most sons did not correct those assumptions so as not to draw attention to same-sex attractions.

“During the talk I kind of went, ‘Oh, okay.’ Just kind of going with it. I didn’t want to tell her I was gay, you know. So I just kind of went with it.”

(Ramos, 18 years old, gay, Latino)

Mortified. Many participants were mortified when parents initiated a sex discussion. The intrusive nature of admonitions and questions caused sons to want to end the conversations quickly.

“We were in the hotel lobby waiting on my mom to get ready and my dad said, ‘Hey, I put a box of condoms in the front of your suitcase. Make sure you put it somewhere that you know where it is.’ It was awkward and I was thinking, ‘Please stop talking.’”

(Bentley, 20 years old, gay, Asian)

Sons were also embarrassed or mortified by sex communication when parents wanted to discuss an overwhelming amount of information or when they encouraged sons to engage in gendered activities or behavior such as viewing heteronormative SEM.

“At various points my mom would ask about my sexual activities and say, ‘You should masturbate,’ and I was like, ‘Mom, why are you talking about this?’ She would also encourage me to… I don’t want to say ‘ogle’ women. That’s a bit extreme, but she’d go, ‘Check out the rack on that one over there,’ like that sort of thing, and I would be like, ‘Mom! Stop!’”

(John, 18 years old, bisexual, Latino)

Isolated. The lack of discussion at home regarding non-heterosexual orientations caused some of the participants to feel isolated. Some GBQ sons wished that parents offered inclusive information about same-sex attractions.

“For me, when [being gay] was not talked about in particular, it made me feel very isolated from my own parents. I expected that from the school, because those people don’t really know me. But from my own parents I expect a little bit more in terms of the nuances of understanding that. I think it’s very important that that is talked about and to make that connection with their children regardless of how parents identify.”

(Alex, 19 years old, gay, Black)

Some sons added that they felt excluded at home when parents ignored early adolescent behavior inconsistent with heterosexual norms (i.e., being repeatedly caught looking at same-sex male pornography). For sons, any inclusion of their sexual orientation during family conversations would have made them feel acknowledged.

“Ever since I told them [about being bisexual], they’ve avoided the subject. My dad still is kind of uneasy about it ‘cause [of] his religious beliefs. And my mom, she totally accepts me for who I am, but we never talk about it. At dinner they have conversations asking my brother about his girlfriend and stuff they do, and I’m just sitting there thinking, ‘I wish you would ask questions about my boyfriend.’”

(Gregory, 16 years old, gay, White)

Offended. After disclosure of sexual orientation, many parents focused on HIV-related issues during sex communication. Most sons found this focus offensive, as it communicated parental reliance on stereotypes about sexual minority men rather than confidence in their own son’s judgment. Parental concern over sons’ future health felt exaggerated and implied a lack of trust regarding their capacity to make safe sexual health choices.

“Every once in a while I’ll get a question about HIV. I feel like they have learned to stop asking because I once was like, ‘Whatever. Of course I know. I do research on this! You think I don’t know what my own risk factors are?’ So I prefer not to talk about my own sex life with them.”

(David, 20 years old, gay, White)

Also, many sons recalled initial sex communication after disclosure as offensively intrusive. The participants viewed themselves as young adults by the time most of these GBQ-specific talks occurred, and inquiries about their sexual history were seen as inappropriate.

Dismissive. Many participants described not paying attention during sex talks, especially when parents assumed they were heterosexual. Many felt the information being covered was not new, as they had learned about it from friends and online sources. Often because of their early and easy access to online sources, including SEM, participants felt they knew enough about sex topics and were more knowledgeable than parents.

“When [my dad] started the conversation I was definitely giggling and saying, ‘Yes, I know all these things.’ I had already known a lot of the topics. I had known what a condom was, how to put it on. I’m not going to say that I knew in detail what gonorrhea was or syphilis was or the other STDs were, but I had a general idea that unprotected sex leads to STDs. I don’t know if I had full understanding of what HIV was, but I had a very basic understanding that it leads to AIDS. So I was thinking, ‘Oh, LOL. My dad is telling me these things that I already know.’ I went, ‘I already know this, dude. Why are you talking to me about it?’”

(Ian, 20 years old, gay, Asian)

### 3.5. Sex Communication Functions for Sons

Despite their generally negative experiences with sex talks, participants viewed sex communication with their parents as a process that served specific functions. From their stories, participants used sex communication to *seek answers*, to *gauge parental opinion and acceptance*, to *keep parents informed*, to *educate parents*, and to *maintain a relationship* for future support.

To seek answers. When sons were younger, they talked to parents when seeking answers to questions of a sexual nature and viewed parents as an accessible source of information. Looking back to before adolescence, participants saw parents as arbiters of reliable information. Participants recalled that they did not have as many qualms about broaching sex-related questions with parents compared to when they were older.

“I had heard the term ‘eating out,’ and then I asked my mom, and she was like, ‘Oh it’s like, it’s like licking a woman’s clitoris.’ I’m pretty sure that’s how that conversation went in the sense of me being curious about sex.”

(Michael, 20 years old, gay, Latino)

To gauge parental opinion and acceptance. Depending on how sons’ self-identified at the time of sex talks, they listened to parents’ words for evidence of parental views related to LGBTQ issues.

“He was this kid in my neighborhood, and we hung out a lot. We were just joking around one day, and he said, ‘I bet you won’t put me as your screen saver.’ And I was like, ‘I bet you I will,’ so I did. But he didn’t know I was bisexual at that time. He just thought we were joking around and having fun. So I did. And then my mom took my phone one day and she was like, ‘Why is this guy on your phone? Who is he?’ She didn’t know him. I was like, “It was just a joke. We were just joking around,” because it really was just a joke. And she was like, ‘No. Normal kids don’t joke like that.’ And she was like, ‘Do you have something to tell me?’ And I immediately said, ‘No, I have nothing to tell you. There’s nothing wrong. What are you talking about?’ And I got really agitated and really irritated whenever she would bring up stuff like that. I’d be like, ‘Mom, just leave me alone. You’re making me feel like an awful person. Just leave me alone.’”

(Gauis, 18 years old, bisexual, Black)

Even if they pretended to be inattentive, GBQ sons who were still figuring out their identities during adolescence were actually listening for clues about how accepting their parents would be regarding same-sex attraction. For others, sex communication was a way to gauge how much parents may have changed their opinions about LGBT issues since their disclosure.

“[My dad] was really upset one night and just sorry for what he did. He told me how sorry he was for treating me really bad. That he wasn’t OK with [me being gay], but he wanted the best for me. And he told me to be safe with my partner. And that’s the only time he said something good. He told me to be safe. He didn’t specify, he just told me to be safe. To take care of myself in different aspects—mental, physical, and all those things. I pretended I was not listening, but I was really attentive. I pretended I wasn’t listening but… those words, they’re going to stay here [gestures with a fist to his heart]. That’s the only time he told me.”

(Tilapia, 19 years old, gay, Latino)

To keep parents informed. Sons reported that sex communication enabled them to keep parents informed of details about their lives. Among the 26 participants whose parents knew about their sexual orientation, the sex communication process enabled them to share who they were dating and even what behaviors they engaged in.

“I feel like a lot of LGBT kids do want to have that kind of sex talk. A lot of them do want to discuss or be able to talk to their parents about sex. I feel like a lot of people want to be more open [with their parents] about who they’re dating and want to be more comfortable talking about stuff like that.”

(Ricky, 20 years old, gay, Latino)

In one case, because a son wanted his relationship to be viewed on the same level as his siblings, he volunteered information.

“Before we were dating, I told them. I was just sitting there at the dinner table, and I just told them, ‘I’m talking to this guy and I like him. I don’t know if we’re gonna date or not but I like him.’ And they were just kinda like, ‘Okay.’”

(Gregory, 16 years old, gay, White)

To educate parents. Sons viewed sex communication as a means to fill their parents’ knowledge gap about issues concerning the LGBTQ community. After disclosure, the main reason sons initiated sex communication was to offer insights about their sexual orientation. Participants recalled how talking about sex allowed sons to clarify certain ideas for their parents, such as the fact that bisexuality is not “just a phase.” Participants felt it was important to address parents’ knowledge gap through sex communication to help parents be more comfortable with the idea that their sons self-identified as GBQ.

“[My parents] used to make small comments basically making fun of gay people calling them names and stuff like that. And my mom was sick for a full year, and she’d stay around the house a lot, and we’d talk a lot ‘cause my dad was at work. We talked about different things of that nature and just generally about how these kinds of views really aren’t that good to have. She basically listened, and she pretty much came to agree with me. She felt the same way I did ‘cause she told me a lot of things she said were things she heard while she was growing up and just repeated, because she thought it was true because she’s heard it from people around her. I think she’s pretty good about stuff like that now.”

(Tyler, 18 years old, queer, Black)

This was especially true for sons whose parents initially had a difficult time accepting their sons’ identity.

“He [My dad] doesn’t think gay people exist. He thinks that people are faking it. I’ve tried to change that, have talks with him, but he’s just fricking conservative. I mean, I can ignore it for a while until it really gets to me. And then I yell at him, ‘I’m gay. Why don’t we talk about it? How come you keep asking me if I have a girlfriend yet? It’s obvious you don’t believe me or something!’ And then I’d explain some more and he’d go, ‘Okay.’”

(Dan, 20 years old, gay, White)

To sustain parent-child relationship. Sex communication was also viewed as a means to ensure a continuous and open relationship with parents, especially in case sons experienced difficulties and needed parental support in the future.

“I feel like communication is key to have a healthy relationship. I wish I had conversations with my parents, but I didn’t. I wish I did. If there were conversations about sex then the child would feel more comfortable about it and wouldn’t be so scared. I just feel like it would help the parent-child relationship, as well. So if something happened to the child, or say a son gets a boyfriend and there’s this huge issue that goes on, but the child doesn’t feel like he can talk to his parents or something. You just need to be able to have that communication and make it okay that the parent knows and the child knows and have it be open.”

(Charles, 19 years old, gay, Latino)

Many participants anticipated and experienced turbulent relationships and viewed the ability to discuss these previous and potential future issues with parents as crucial.

“There’s an idea of making them [parents] feel comfortable talking about your partners. Also, you don’t have to talk to them about every Friday night hook-up you’ve had, but sometimes you do have hook-ups and they can be very scary, and you don’t remember who you’ve had sex with. Did you have unprotected sex? I’m just thinking hypothetically. I could very easily have hooked up with someone, and I don’t know what their status was. Your parents are supposed to be your outlet. I think it could be very helpful to have that kind of outlet and say, ‘I think I made a mistake and for mental support, I need you,’ or, ‘Guide me.’ I think it is very, very important.” (Ian, 20 years old, gay, Asian)

### 3.6. Perceived Sex Communication Functions for Parents

Though we only collected data from GBQ sons and not their parents, our participants also discussed how they understood the functions of sex communication for their parents. Sons perceived that their parents used sex talks to *educate them*, to *dictate behavior*, and *to augment previous heteronormative discussions*.

To educate sons. At its core, sons viewed sex communication as a process parents used to educate children on a variety of health and sex topics. During sex communication, the traditional notion was that parents explained and sons tried to understand the topics. Sometimes mothers would provide additional information or a counterpoint to information that sons had received from their fathers or the media.

“If there was a sex scene then [my mom] would kind of explain it to me. She would be like, ‘Oh, that’s not really realistic, don’t try that, you’ll probably hurt yourself!’ My mom specifically told me to never watch porn because she said it would give me unrealistic ideas of what sex looked like. So I never—I can say to this day—I still haven’t really seen porn.”

(John, 18 years old, bisexual, Latino)

Many parents followed up sit-down conversations with repeated check-ins to make sure sons understood what they talked about and to share parental values.

“It was interesting because when my mom implied that she still expected me to be safe with dating and sex [after I came out], she used some phrasing like, ‘I still expect you to be smart about this.’ And I thought it was definitely reasonable, but I also remember thinking, ‘I don’t remember you ever telling me about this expectation you had before!’” (laughing)

(Jonathan, 18 years old, gay, White)

To dictate behavior. Prior to sons coming out, sex communication was a way parents could dictate how sons should behave. This rule-setting function mostly came in the form of verbal instructions. Rule setting was reinforced through gendered scripts that reinforced heterosexual couplings as normative, which sons remembered as frustrating.

“Right before I left home, I received the university directory for incoming freshmen and she said, ‘Pick out all the girls you think are pretty.’ I was like, ‘No,’ and she was like, ‘I just wanna know,’ and I was like, ‘I’m not going to pick out girls for you, I’m sorry.’ So she just made weird comments and gave me that stink face or whatever.”

(Charles, 19 years old, gay, Latino)

The most frequently repeated rule from parents was that sons should abstain from sex before marriage.

“He [dad] mentioned that sex was pleasurable, and you only share it with people you’re married to. ‘Premarital sex is a huge sin and really terrible. Be sure to wait until you get married, and promise me you’re not going to have sex.’ I was like, ‘Sure, okay, I guess.’”

(Alex, 16 years old, gay, Black)

To augment previous heteronormative sex communication. Given that many participants acknowledged that they consciously withheld information about their sexual orientation from parents, sex communication after disclosure was remembered as a way parents augmented previously heteronormative sex talks. After disclosure, many parents provided subsequent reminders for sons to be safe with varying mentions of health concerns prevalent among GBQ men, such as HIV and other STIs. Sons recalled how upon learning of their sexual orientation, many parents revisited some topics and made sure to talk more about topics in a same-sex context. Revisiting sex communication was a way for parents to retrospectively cover essential topics they thought GBQ sons needed to know.

“When I told them I was gay, my mom all of a sudden was, ‘Oh, there’s always that stigma or association of being gay with AIDS or STDs,’ and it kind of opened the door to her talking to me more. And not just sex itself, but relationships and that kind of stuff. Some random news interview came up the other day on TV about a guy who was abused by his spouse, his male spouse. And she was like, ‘Watch this. Don’t get in a relationship where you’re being abused.’”

(Ramos, 18 years old, gay, Latino).

## 4. Discussion

Overall, parent-child sex communication between GBQ youth and their parents in this study reflected findings previously reported in the literature with presumably heterosexual participants. However, young GBQ men in our sample faced additional challenges because the timing of these talks often occurred after they started engaging with SEM and prior to coming out to their parents. Additionally, these discussions were largely heteronormative and included gender policing messages, such as enlisting sons to comply with, and participate in, traditionally heterosexual masculine activities [34]. Our findings suggest that sexual identity milestones, such as the coming out process, complicates the ways that parental sex communication may buffer or brake GBQ sons’ use of SEM and its impact on their sexual development and safety.

Conversation prompts and parental approaches in this analysis are similar to findings reported by heterosexual populations. For example, the four primary prompts to sex communication we identified—son-initiated conversations, physical maturation and social milestones, sharing family stories, and capitalizing on other teachable moments—have been well-documented from studies with presumably heterosexual participants [35]. Similar to what has been reported for heterosexual youth, most of the approaches to sex-communication and content discussed with GBQ sons were consequence-oriented [36]. Parents presented sex negatively, communicating strict rules about sex, and overemphasizing the health and social risks of engaging in sex at an early age [37]. These parental approaches reflect attempts to “brake,” or slow down sons’ engagement in sexual activity and use of SEM. However, our findings show that the timing of parent-child sex communication with GBQ sons may be occurring too late in their sons’ sexual development and exploration to be an effective “brake.” That is, sex communication with GBQ sons occurred years after their initial exposure to SEM (10.9 years), with sexuality-sensitive talks occurring even later when this communication was triggered by disclosure to parents (15.4 years).

In addition to the late timing of parental sex talks, GBQ sons also reported that the overtly heteronormative tone and content of the talks discouraged them from relying on their parents for sexuality-relevant information. This may help explain GBQ sons’ reliance on the internet and SEM to access sexuality information [5], and highlights the anticipatory stress these youth face when hiding their same-sex attraction from parents who assume heterosexuality. Many parents anticipated GBQ sons reaching heteronormative milestones at specific times. For example, inquiries from parents about which girls sons found attractive or were dating communicated heteronormative and gendered expectations. GBQ sons had to learn to respond to these questions in ways that would not draw attention to their same-sex attractions. At times, when parents addressed their sons’ sexual development, they enacted gender policing, which participants experienced as mortifying, isolating, and offensive. These negative reactions by youth reflect similar findings that have identified parent-child communication as discouraging, dismissive or indifferent of sons’ sexual orientation [38]. Concealing aspects of one’s emergent identity and experiencing gender policing has been linked with substance use and psychological distress among sexual minority males [34]. These parental approaches, therefore, are likely to limit the potential for valuable sex communication and have lasting implications for future communication with parents, providers, or potential partners.

Our findings characterize sex communication as a process that often occurs late in GBQ sons’ sexual development, with inclusive sex talks only happening post-disclosure. These conversations then are more reactionary than preventative. Parents of heterosexual adolescents also often initiate conversations about sexual health mostly after children start having sex [35]. For GBQ sons, sex communication is further complicated by sexual identity concealment and earlier and increased exposure to SEM. As a result, parental sex communication with GBQ sons may function more as a “buffer” to the use and impact of SEM. After disclosure, parents were able to use sex communication to clarify and correct what they considered to be incorrect information, provide relevant and specific safety information about same-sex sexual behavior, and affirm their expectations that sons have responsible sexual relationships. Our findings lend support to the adaptive self-organization nature of families with LGBT children, which highlights the malleable nature of the family unit that would benefit from appropriate interventions during moments of disequilibrium and reorganization [39].

Despite having mostly negative initial reactions to sex talks, sex communication was nevertheless viewed by GBQ sons as a functional process that can ultimately have positive implications for their parents’ level of knowledge about GBQ-specific issues. Participants viewed sex communication as a crucial opportunity to bridge knowledge gaps prior to, during, and following their disclosure as GBQ. Young GBQ men valued their relationships with parents [28]. However, participants also highlighted what they wished for in their conversations about sex with parents, including to be able to ask them questions, be affirmed, and to talk openly about their relationships and sexual lives. Given the prominence parents have in their sons’ lives, these functions point to opportunities that parents can leverage to improve the inclusivity and relevance of the sexual health information they share [12].

The nascent research on sex communication between parents and GBQ sons reveals an opportunity for parents to be more active in the provision of sexual health information at a behavior-defining phase. Two strengths of this work include our thorough qualitative exploration of the experiences of GBQ youth with PCSC that addresses the inherent limitations of cross-sectional studies and the inclusion of GBQ youth who are still living at home with their parents. Our results advance what is known about parent-child sex communication for GBQ adolescents and point to questions for future research. Future work should center parents’ perspectives on sex communication and their informational needs for this dyadic process with GBQ adolescents. Furthermore, longitudinal and observational studies may also provide more detail about the long-term impact sex communication has for both parents and GBQ sons. Additionally, focused examination of the sociocontextual and cultural factors that uniquely impact families from different racial/ethnic backgrounds is in order to begin identifying ways that future interventions may be tailored. Sociocultural factors influence sexual and gender identities and family dynamics, which demands going beyond one-size-fits-all intervention approaches [39].

Our findings should be considered in light of study limitations. Our sample consisted mostly of young men who were also involved with local LGBT organizations. This may limit the generalizability of the results to other, less community-engaged GBQ youth. Further, the size of our sample limited our ability to appreciate any variations in the findings between racial or ethnic groups. This limitation is important to note because macro-level factors such as one’s racial and ethnic identity or a family’s religious affiliation strongly influence discussions about sex in the home [40]. Additionally, the majority of our participants came from supportive families whose parents were accepting of their sons after they disclosed their sexual orientation. Many GBQ youth experience parental rejection and only one participant in our sample reported this experience. Future studies should explore the parent-child sex communication experiences of youth whose family relations were highly strained or were severely disrupted after disclosure. Furthermore, findings from this study may be used for future work including measurement development specific to families with sexual and gender minority children, as most communication scales currently used in the field of family sexuality and communication studies were developed and validated with presumably heterosexual parent-child dyads. Future research may also leverage our findings to inform large-scale population-level quantitative surveys to examine relationships between PCSC and GBQ health outcomes and for intervention development [23].

## 5. Conclusions

Results from this study add to the case for parent-child sex communication as a potential protective factor for GBQ youth. Our study revealed that parent-sex communication prompts, content, and functions for GBQ sons were similar to that reported in samples of heterosexual youth. However, existing evidence-based parental resources for sex communication with heterosexual youth need to be systematically adapted and tailored to the needs of GBQ youth. Our findings also support the importance of focusing on communication (including prompts, content, and functions) in public health interventions. These interventions should emphasize timing and approaches to mitigate the awkward, uncomfortable, and embarrassing conversations between parents and GBQ youth. Adolescence is a critical period that often includes GBQ sons’ first disclosure of their sexual orientation and exploration of SEM. Addressing parents’ knowledge gaps about their GBQ sons and SEM, along with developing their capacity to initiate and sustain affirming and meaningful parent-child sex communication, has the potential to normalize their son’s emergent GBQ selves, provide positive social messages about sexual health, and contribute to developing healthy sexual relationships and practices.

## Data Availability

The data presented in this study are available on request from the corresponding author.
